# Citrullination and the protein code: crosstalk between post-translational modifications in cancer

**DOI:** 10.1098/rstb.2022.0243

**Published:** 2023-11-20

**Authors:** Koyo Harada, Simon M. Carr, Amit Shrestha, Nicholas B. La Thangue

**Affiliations:** Laboratory of Cancer Biology, Department of Oncology, University of Oxford, Old Road Campus Research Building, Oxford OX3 7DQ, UK

**Keywords:** protein, peptidylarginine deiminase, citrullination, cancer, methylation, post-translational modifications

## Abstract

Post-translational modifications (PTMs) of proteins are central to epigenetic regulation and cellular signalling, playing an important role in the pathogenesis and progression of numerous diseases. Growing evidence indicates that protein arginine citrullination, catalysed by peptidylarginine deiminases (PADs), is involved in many aspects of molecular and cell biology and is emerging as a potential druggable target in multiple diseases including cancer. However, we are only just beginning to understand the molecular activities of PADs, and their underlying mechanistic details *in vivo* under both physiological and pathological conditions. Many questions still remain regarding the dynamic cellular functions of citrullination and its interplay with other types of PTMs. This review, therefore, discusses the known functions of PADs with a focus on cancer biology, highlighting the cross-talk between citrullination and other types of PTMs, and how this interplay regulates downstream biological events.

This article is part of the Theo Murphy meeting issue ‘The virtues and vices of protein citrullination’.

## 1. Introduction

**Figure 1.**
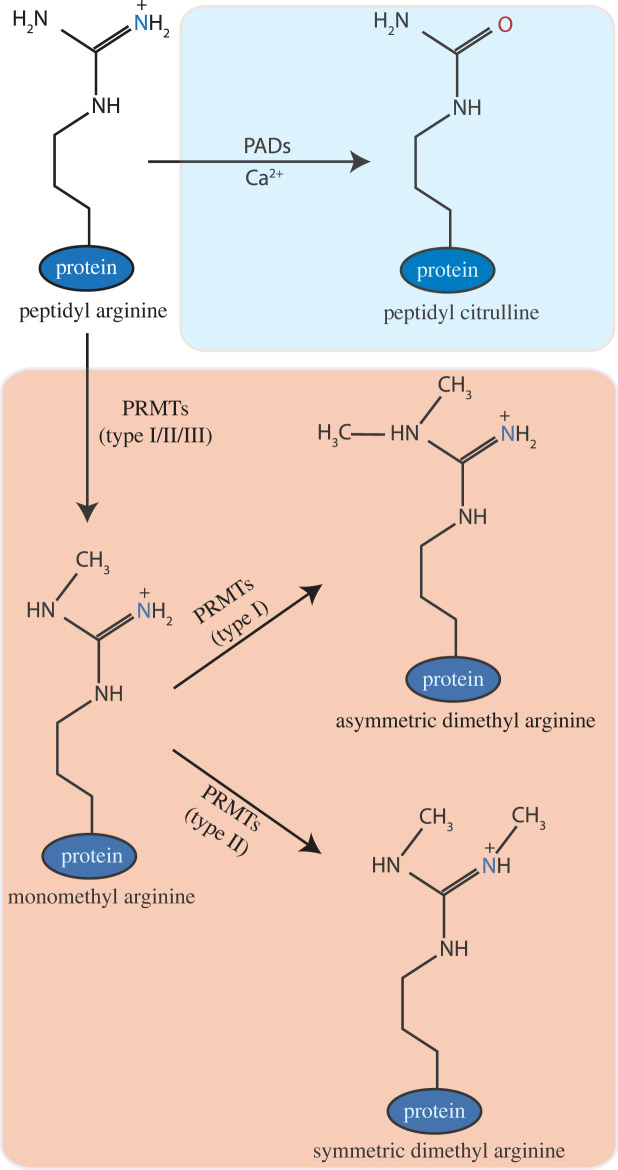
Citrullination of peptidyl arginine by PADs and methylation by protein arginine methyl transferases (PRMTs). Enzymatic conversion of peptidyl arginine into peptidyl citrulline catalysed by calcium-dependent peptidyl arginine deiminase (PADs) is background shaded in blue, while conversion of peptidyl arginine into mono methyl arginine, asymmetrical dimethyl arginine or symmetrical dimethyl arginine mediated by PRMTs is background shaded in orange.

Post-translational modifications (PTMs) have been heavily implicated in cancer and other diseases, due to their importance in the functional expansion of target proteins. Out of more than 200 modifications identified [1], citrullination is attracting increasing interest [2]. The modification is unique as it does not involve the addition of a small chemical group like other PTMs, but rather the direct conversion of an arginine residue (R) into citrulline. Specifically, citrullination involves the hydrolysis of a guanidinium group of an arginine residue, leading to the production of urea and a loss of positive charge and two potential hydrogen bonding donors [3,4] (figure 1). As a result, citrullination may change the target protein's activity, or modulate its interaction with other protein(s) and nucleic acids.

Protein citrullination is catalysed by a family of calcium-dependent enzymes called peptidylarginine deiminases (PADs). There are five PADs identified in the human genome, including PAD1, PAD2, PAD3, PAD4 and PAD6. The enzymes exhibit highly conserved structures, and their genes are known to be clustered at chromosome position 1p36.13 [3]. They also demonstrate unique patterns in terms of tissue-specific expression and target substrates [5]. No ‘de-citrullinase’ enzyme that removes or converts peptidyl citrulline back to native peptidyl arginine has yet been identified [2], and thus citrullination is currently considered an irreversible modification on arginine residues.

Under physiological conditions, PADs are usually regarded to be inactive since they are thought to require a high concentration of calcium ions (10^3^–10^4^ nM) for activation [6,7], and physiological intracellular calcium ion concentrations are usually maintained around 100 nM [8]. Not surprisingly, PADs are, therefore, proposed to be involved in biological events in which the intracellular concentration of calcium exceeds typical physiological levels, such as during apoptosis and terminal differentiation [9,10]. However, several reports suggest PADs are involved in gene regulation under physiological conditions; for example, about 10% of histones in HL60 granulocytes are citrullinated [11]. This suggests that there may be a mechanism that circumvents the calcium requirement for PAD activity, or supra-physiological pockets of high calcium concentration exist in cells.

Another physiological function of PADs involves the formation of neutrophil extracellular traps (NETs) by cells of the immune system, such as neutrophils, monocytes and macrophages [12,13]. During the process of NET formation (often referred to as ‘NETosis’), both the nuclear and cytoplasmic membranes of the cell break down, resulting in the release of modified chromatin, bacteriocidal proteins and other lysosomal constituents including neutrophil elastase (NE), myeloperoxidase (MPO), cathelicidin and cathepsin G into the extracellular environment [14–16]. Given that NETosis primarily involves immune cells, it is considered as a unique form of programmed cell death that occurs as part of an immune response against pathogens such as bacteria and viruses [12,13]. PAD4 activity was shown to be an important regulator of NETosis, since PAD4-dependent citrullination of histones is required for chromatin decondensation and release, and neutrophils isolated from PAD4 knockout mice do not form NETs [12,13,17]. In the past decade, interest in NETosis has increased, since this process was discovered to be deregulated in numerous inflammatory diseases and several types of cancer [18,19]. Much still remains to be elucidated about the contribution of NET formation to the progression of pathological conditions, and the contribution of PAD enzyme activity to these processes requires further investigation [20].

Deregulated PAD activity and abnormal levels of citrullination are found in various inflammatory diseases including rheumatoid arthritis (RA), systemic lupus erythematosus (SLE), Alzheimer's disease and cancers [21]. RA is the most studied example in terms of citrullination, where increased activities of PAD2 and PAD4 are observed [22]. Citrullinated proteins present in the synovial fluid of RA patients are thus a known hallmark of the disease, and these modified proteins are thought to lead to the production of anti-citrulline peptide antibodies (ACPA) that intensify the auto-immune and inflammatory response experienced by patients [23,24]. Autoantibodies generated against peptidyl citrullines are found in around 70% of RA patients and are often used for diagnostic and predictive purposes, whereby the presence of ACPA at early diagnosis is correlated with disease progression and joint damage in human patients [23]. The molecular mechanisms underlying how ACPA contribute to the development of RA are not fully understood, but several *in vitro* studies indicated that ACPA were involved in multiple inflammatory responses including the production of TNF-α, NET induction and promoting osteoclastogenesis [24]. Furthermore, PAD4 has been identified as an antigenic target in RA, with reports suggesting the presence of anti-PAD4 autoantibodies in a subgroup of RA patients [25–27]. Notably, it has been observed that the binding of these autoantibodies to PAD4 enhances its enzymatic activity by reducing the sensitivity to Ca^2+^ ions, thus offering a potential mechanism by which PAD4 contributes to the pathogenesis of RA [28].

Deregulated PAD activity has, therefore, been shown to play an important role in inflammatory diseases such as RA, and significant effort has been devoted to investigating the potential clinical use of PAD-specific inhibitors for these conditions. Some promising pre-clinical results have already been reported for the pan-PAD inhibitor Cl-amidine, and its modified derivative BB-Cl-amidine, which were shown to reduce the severity of several inflammatory conditions in murine models for collagen-induced arthritis (CIA) [29], colitis [30] and lupus [31]. Some other PAD isozyme-specific inhibitors, including AFM-30a (PAD2) and GSK199 (PAD4), had similar effects in mice models [32–34].

Given that the abnormal expression of PADs is also found in cancer, and inflammation is a hallmark of cancer [35], investigating how PAD activity contributes to cancer progression and pathogenesis is an area of current research. In addition, there is growing interest in the role that citrullination plays in the broader context of its interplay with other PTMs, particularly epigenetic marks implicated in the progression of cancer. We will discuss recent studies regarding PAD activity and relevance in cancer, as well as its cross-talk with other PTMs as part of a wider code that regulates protein activity.

## 2. Citrullination in cancer

### (a) Expression of peptidylarginine deiminases in tumour tissues

An increasing body of evidence now indicates the important roles that PADs undertake in cancer progression. Since PAD2 and PAD4 are the most widely expressed members of the PAD family, most studies in cancer focus on these two enzymes, while the other family members have to date not been extensively researched [2,36]. The physiological tissue distribution and deregulated expression of these two PADs in cancer are summarized in table 1.

**Table 1.** Tissue distribution and over-expression of PADs in cancer.

PAD isotype	tissue distribution	over-expression in cancer	references
PAD2	salivary gland, brain, pituitary gland, bone marrow, spinal cord, uterus, skin, spleen, pancreas, kidney, skeletal muscle, immune cells	gastric (tissue and blood), liver (tissue and blood), colorectal (tissue and blood, though decreased expression in tissue also reported), oesophagus; (tissue and blood), breast (tissue and blood), skin	[37–42]
PAD4	immune cells (neutrophils, monocytes, macrophages), brain, pituitary gland, uterus, joints, bone marrow	gastric (tissue and blood), liver (tissue and blood), ovarian (tissue and blood), colon, endometrial, hepatocellular, lung, bladder	[43–50]

Over-expression of PAD4 has been reported in numerous cancer types [43–50], in contrast to benign tumours and non-tumour inflamed tissues that do not exhibit high levels of PAD4 expression, suggesting its association with cancer progression and its potential as a cancer drug target [43]. Increased levels of PAD4 were also detected in the blood of patients suffering from a variety of cancer types, including breast, lung, colon, ovarian and prostate cancers [44]. PAD2 was shown to be deregulated in multiple tumour types, with increased expression observed in breast, liver, lung, ovarian and prostate cancer tissues, as well as in the blood of cancer patients [37–40,51]. The role of PAD2 in colorectal cancers appears complex, with both increased and reduced expression of the enzyme having been reported [41,42].

### (b) PAD4-mediated citrullination and gene regulation in cancer

PADs play a regulatory role in gene expression at the level of transcription, classically regarded to occur through citrullination of histone substrates [52,53]. This is mainly mediated by PAD4, which is the only PAD member with a nuclear localization sequence (NLS) [54]. PAD4 citrullinates multiple arginine residues of histones H3 and H4 and thus could compete with other enzymes that introduce modifications onto these same residues, such as arginine methyltransferases [52,53]. In addition to histone citrullination, PAD4 is also known to regulate gene expression via citrullination of transcription cofactors or direct modification of transcription factors themselves (including cofactors such as p300, and the transcription factors E2F1 and ELK-1, discussed in §3c) [55–57].

The importance of PAD4-mediated gene regulation in biological processes is well exemplified by the interplay between PAD4 and the tumour suppressor p53. In a p53-dependent manner, PAD4 can be recruited to the promoter region of p53-target genes such as *OKL38*, *p21*, *CIP1* and *WAF1*, where it catalyses histone citrullination that negatively regulates gene expression [58,59]. In response to ultraviolet (UV) DNA damage, the recruitment of PAD4 to the promoter of p53-target genes involved in cell cycle control and apoptosis (such as *p21*, *GADD45* and *PUMA*) was significantly reduced, resulting in activated transcription [60]. This suggests that PAD4 has an important role in providing a repressing mark that acts to regulate p53-dependent cell cycle regulation and apoptosis [60]. However, the role of PAD4 and p53 in the control of apoptosis is not restricted to just gene regulation, since another study indicated that p53- and PAD4-dependent citrullination of histone H4R3 in response to adriamycin (ADR) treatment was important for chromatin decondensation and fragmentation in apoptotic U2OS cells [61]. The H4R3cit mark was localized around the fragmented nuclei of these cells, and H4R3cit expression correlated with the level of apoptosis induction [61]. It was later shown that *PAD4^−/−^* mice are resistant to radiation-induced apoptosis in the thymus, and the H4R3cit mark was associated with smaller tumour size in non-small cell lung cancer tissues [61]. The interplay between p53 and PAD4 in apoptosis regulation is, therefore, intricate, and likely impacted by the type of damage signal received. However, the hypothesis that PAD4 plays an important role in apoptosis is further supported by the fact that maximum PAD4 enzyme activity requires elevated calcium ion concentrations, and calcium ion influx into cells has been implicated in regulating apoptotic signals [62,63]. Since other PADs have not been shown to interact with p53, PAD4-mediated citrullination may play a unique role in p53-mediated apoptosis.

Interestingly, a role for PAD4 in transcriptional activation has also been reported. According to Zhang *et al.* [55], PAD4 was observed to be enriched at the transcription start site of actively transcribed genes in MCF7 breast cancer cells, where it plays a role as a coactivator for a wide range of transcription factors. The study revealed that PAD4 directly targets citrullination of arginine residues on the transcription factor ELK-1, which promotes its subsequent phosphorylation by the kinase ERK2 [55]. It was predicted that phosphorylated ELK-1 then associates more strongly with the histone acetyltransferase p300, leading to enhanced histone acetylation at target promoters and gene activation [55].

In addition to gene regulation, an ever-increasing body of literature suggests that the PAD4-mediated formation of NETs also contributes to tumour progression by promoting metastasis and proliferation in various types of cancer, observed in both animal models and patients [64–67]. For example, a recent study demonstrated that abundant levels of NETs were found in liver metastases of patients with primary breast cancer [68]. Here, NETs were suggested to act as a chemotactic signal to promote metastases, whereby DNA contained within the NET structures was detected by the trans-membrane extracellular sensor protein, CCDC25, which further promotes cell motility through the activation of the ILK-β-parvin pathway [68]. PAD4 is involved in the formation of NETs [17,69,70] and its depletion correlated with decreased NET formation as well as reduced metastasis in murine models of colorectal and breast cancer [71–74].

Unsurprisingly, there is growing interest towards targeting PAD4 and citrullination as a potential therapeutic approach to treat cancer or improve cancer-associated morbidities [18]. For example, renal insufficiency is a frequent cancer-associated toxicity, believed to be caused by the formation of intravascular tumour-induced NETs in the kidneys [75]. Cedervall *et al.* [75] demonstrated that the depletion of NETs by pharmacochemical PAD4 inhibition led to the recovery of renal function in tumour-bearing mice. In addition, reports have indicated that increased levels of citrullinated histone H3 or NET formation are correlated with poor clinical outcomes or progression of cancer [76,77]. However, the underlying molecular mechanisms of NETosis and its role in tumour biology are not fully understood, which is currently an active area of ongoing research.

### (c) PAD2-mediated citrullination and gene regulation

PAD2 is the most widely expressed member of the PAD family [78]. Despite lacking a NLS, both the Ca^2+^-dependent nuclear localization of PAD2 and PAD2-mediated citrullination of histones have been described [79–81].

PAD2 has an important role in castration-resistant prostate cancer (CRPC) progression [82]. This late-stage disease is a major burden for hormonal therapy because the tumour cells are able to proliferate independently of androgen receptor (AR) signalling [83]. Wang *et al.* [82] reported that PAD2 is involved in CRPC progression and tumour cell proliferation under androgen deprivation or castration. They demonstrated that PAD2-dependent citrullination rescues AR from ubiquitin-mediated degradation and promoted its nuclear translocation and target gene-binding [82]. Furthermore, co-targeting of PAD2 and AR with the small molecule inhibitors Cl-amidine (PAD enzyme inhibitor) and enzalutamide (AR signalling inhibitor) was shown to have a synergistic effect on reducing tumour growth, as compared to the treatment of either compound alone in a prostate cancer mouse model [82].

PAD2 expression is also observed in human mammary tissues and is deregulated in mammary carcinomas, especially in the HER2-positive luminal subtypes [37,84]. One study reported that PAD2 is involved in cancer transformation and progression [37], while a second suggested that PAD2-mediated citrullination regulates cell migration in breast cancer cell lines via modulating the expression of cytoskeletal regulatory genes [40]. Another study discovered that PAD2 can regulate gene expression and profileration of breast cancer cells via its ability to target an arginine residue in the C-terminal domain of RNA polymerase II (RNAP2), which facilitates the interaction between RNAP2 and the positive transcription elongation factor b (P-TEFb) complex [85]. Although further mechanistic details need to be acquired, these studies strongly imply that PAD2 is involved in breast cancer progression via gene expression regulation.

In colorectal cancer, however, PAD2 can play a repressive role in cancer progression. PAD2 expression was reported to be downregulated during the early stages of the disease, and its low expression level is correlated with poor prognosis [42]. Funayama *et al.* [86] revealed that PAD2 suppresses the proliferation of colon cancer cells, whereas over-expressed PAD2 caused cell cycle arrest. Another study reported that PAD2 directly regulates Wnt (wingless)/β-Catenin signalling, a critical pathway in carcinogenesis [87]. According to Qu *et al.*, the small molecule nitazoxanide (NTZ), which is a clinically approved antiparasitic and antiviral drug, inhibits this signalling pathway through directly targeting and stabilizing PAD2. Stabilized PAD2 citrullinates β-Catenin and targets the protein for degradation [87], and as a result, NTZ treatment was demonstrated to suppress the growth of colon cancer cells [88].

These discoveries collectively document the multi-functional and tissue-specific aspects of PAD2 activity in tumourigenesis. The significance and potential anti-tumour effects of PAD2-specific inhibitors have been highlighted in multiple pre-clinical studies [89,90], though further research is required to understand the functions of PAD2-mediated citrullination events in the pathology of different types of cancer [87,88].

## 3. Cross-talk between citrullination and other epigenetic modifications

**Figure 2.**
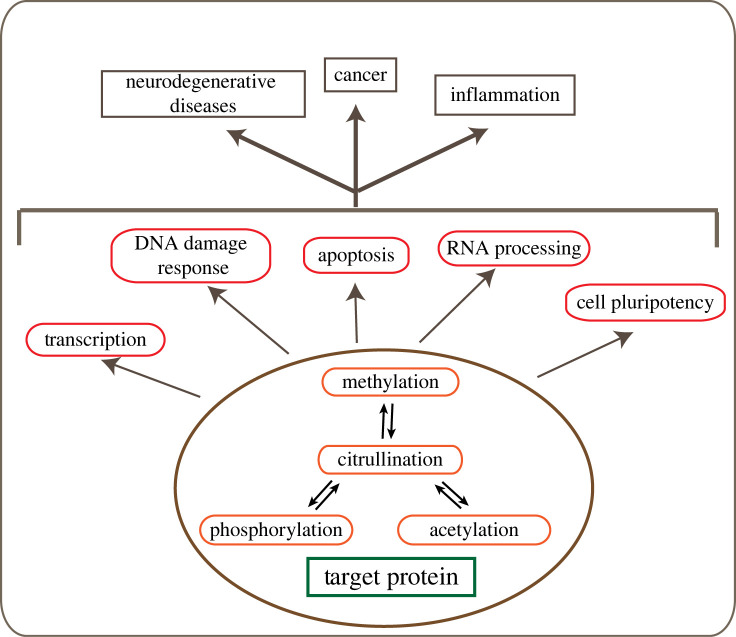
Cross-talk between citrullination and other post-translational modifications. An overview of the cross-talk between citrullination and other post-translational modifications, and its implication in various biological processes associated with neurodegenerative diseases, inflammatory diseases and cancer.

Epigenetic modifications, including those that occur on both histone and non-histone substrate proteins, rarely function alone, but often work together in various combinations to delicately regulate functional outcomes [91,92]. Indeed, citrullination, like other PTMs on proteins, can often influence the generation of other nearby post-translation marks such as methylation and acetylation. This ‘cross-talk’ can have a downstream impact on the recruitment of certain ‘reader’ proteins, which can influence further protein–protein interactions and biological processes such as gene expression (figure 2).

### (a) Citrullination and arginine methylation

In recent years, arginine methylation has garnered particular interest owing to its involvement in a multitude of cellular processes including transcriptional regulation, RNA processing, DNA repair and signal transduction pathways [93,94]. It involves the sequential addition of methyl groups to the guanidino group of an arginine residue to generate mono-methylated (Rme1) and di-methylated arginine, which can occur in either a symmetric (Rme2s) or asymmetric (Rme2a) fashion (figure 1). Such reactions are mediated by a family of enzymes known as the protein arginine methyltransferases (PRMTs) [93,94], which are classified into three groups based on their ability to catalyse the formation of Rme2a (type I PRMTs such as PRMT1 and 4), Rme2s (type II PRMTs such as PRMT5) or only Rme1 (type III PRMTs such as PRMT7) (figure 1) [93,94]. Given their critical roles in numerous biological pathways, deregulated expression of the PRMTs has been connected with multiple types of cancer, as highlighted in table 2.

**Table 2.** Deregulation of PRMTs in different cancer types.

PRMT isozymes	deregulation in cancer	references
PRMT1	over-expressed and aberrantly spliced in leukaemia, breast, lung, colon and bladder cancers	[95–103]
PRMT2	over-expressed in breast cancer	[104,105]
PRMT3	over-expressed in breast cancer	[106,107]
PRMT4 (CARM1)	over-expressed in breast, prostate and colorectal cancer	[108–113]
PRMT5	over-expressed in lung, gastric, colorectal cancer, leukaemia, lymphoma and melanoma	[114–121]
PRMT6	over-expressed in lung and bladder cancer	[97,122]
PRMT7	over-expressed in breast cancer	[123]
PRMT8	somatic mutation in ovarian, skin and colon cancer	[124]

**Figure 3.**
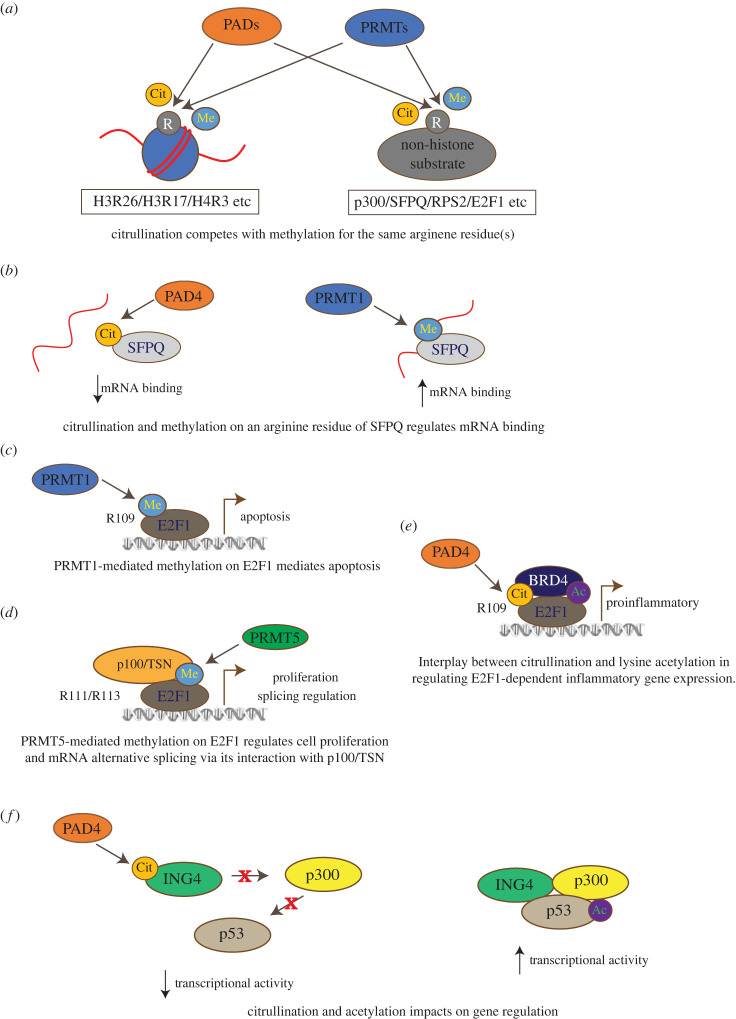
Examples of cross-talk between citrullination and other PTMs. (*a*) PADs and PRMTs compete for the same arginine residues for citrullination and methylation. As a result, the interplay between PAD and PRMT activity can modulate the downstream activity of substrate proteins. (*b*) Citrullination on an arginine residue of splicing factor proline- and glutamine-rich (SFPQ) protein by PAD4 decreases its binding to mRNA, whereas methylation by PRMT1 on the same arginine residue increases its association with mRNA. (*c*) PRMT1-mediated methylation of E2F1 at arginine residue 109 promotes the expression of E2F1-target genes involved in apoptosis, while (*d*) PRMT5-mediated methylation of E2F1 on arginine residues 111 and 113 regulates the expression of genes involved with cell proliferation. In addition, the recruitment of p100/Tudor staphylococcal nuclease (TSN) to the methyl marks engages E2F1 with the regulation of alternative RNA splicing. (*e*) PAD4 mediates E2F1 citrullination on arginine residue 109, which enhances the interaction between BRD4 and acetylated E2F1 at the promoters of inflammatory genes. (*f*) ING4 enhances p53 transcriptional activity by recruiting the p300 acetyl transferase and promoting acetylation of p53. However, PAD4-mediated citrullination of ING4 disrupts the interaction with p53 as well as the recruitment of p300, thereby reducing the level of acetylation and leading to reduced transcriptional activity.

Perhaps the most obvious form of cross-talk between citrullination and other PTMs would be that observed with arginine methylation since both modifications can theoretically compete for the same arginine residue in a substrate protein (figure 3*a*). Indeed, a number of arginine residues on histone tails have been identified as targets for deimination, and these same residues can also be targeted by methylation. For example, PAD4 targets the conversion to citrulline on H3R26, H3R17 and H4R3 [52,53,125], while these same sites can be targeted for methylation by PRMT4/CARM1 and PRMT1, respectively [126–130] (table 3).

**Table 3.** Examples of crosstalk between citrullination and other PTMs on target substrates.

substrate	sites for crosstalk	biological consequences	references
histones	H3R2/8/17/26 and H4R3 targeted by PRMT1/4 for **Me** and PAD4 by **Cit**.	**Me** and **Cit** target histones in the promoters of genes such as *pS2*, *CTCF* and *OKL38*, to regulate gene expression positively or negatively.	[52,53,58,127,131–133]
	H3R26 targeted by PAD2 for **Cit**. H3K27 targeted by EZH2 for **Me**.	H3R26Cit can drive the expression of ER-responsive genes and reduce the activity of EZH2 against H3K27. H3K27Me is a repressive mark and inhibits the binding of PAD2 to H3. During the establishment of naive pluripotency, SMARCAD1 is recruited to H3R26Cit, which represses H3K9me3 marks.	[80,81,134]
	H3R8 targeted by PAD4 for **Cit**. H3K9 targeted by some SET domain lysine methyltransferase for **Me**.	H3K9Me3 at the promoters of pro-inflammatory genes, such as *TNFα* and *IL8*, recruits HP1*α*, which leads to reduced gene expression. Neighbouring H3R8Cit can interfere with this interaction, resulting in de-repression of these genes.	[135]
p300	C-terminal R2142 residue targeted by PRMT4 for **Me** and PAD4 for **Cit**.	**Me** promotes and **Cit** inhibits the interaction between p300 and GRIP, affecting the transcriptional coactivator function of this complex.	[57]
SFPQ	Several N-terminal R residues targeted by PRMT1 for **Me** and PAD4 for **Cit**.	**Me** promotes and **Cit** inhibits the association between SFPQ and its target mRNA.	[136]
RPS2	R residues in N-terminal RG-repeat region targeted by PRMT3 for **Me** and PAD4 for **Cit**.	**Me** of RPS2 may regulate the biogenesis of ribosomes in yeast.	[137–140]
RGG motif proteins	R residues of RGG motifs of various proteins such as FET proteins and hnRNPA1 targeted by PAD4 for **Cit** and PRMTs for **Me**.	**Me** of splicing factors regulates RNA-binding affinity.	[141–152]
E2F1	R109 targeted by PRMT1 for **Me** and PAD4 for **Cit**. R111/113 targeted by PRMT5 for **Me** and antagonizes R109 **Me**.	Each PTM channels E2F1 into a distinct biological pathway; R109Me to apoptosis, R111/113Me to proliferation, and R109Cit to pro-inflammatory gene expression.	[56,114,153]
	R109/127 targeted by PAD4 for **Cit**, which flank the K117/120/125 targeted for Acetyl.	E2F1–BRD4 interaction is primarily dependent on **Ac**, and further enhanced by **Cit**.	[56]
ELK-1	Protein targeted by PAD4 for **Cit** and its S383 is targeted by MAP kinase pathway for phosphorylation.	**Cit** of ELK-1 facilitates the subsequent phosphorylation.	[154,155]
ING4	ING4 is targeted by PAD4 for **Cit**. p53 is targeted by p300 for **Ac**.	PAD4-dependent **Cit** of ING4 inhibits the recruitment of p300 to p53, leading to reduced p53 **Ac** and downstream gene suppression.	[156–158]

Since citrullination by the PAD family of enzymes effectively deiminates the peptidyl arginine residue by removing one of the two terminal amino groups to convert it to citrulline, this modification would effectively block recognition and conversion to methyl arginine by the PRMTs, and therefore retain the residue in a chemically demethylated state. However, the question of whether methylated peptidyl arginine could be converted to citrulline was complicated by early reports proposing that PAD4 might act to reverse methylation by hydrolyzing methylated arginines in a ‘demethylimination’ reaction [53,125]. Such a reaction may not represent true reversal of methylation, since citrulline would result rather than conversion back to arginine, but it would serve an analogous function. This hypothesis was supported by experiments in cells which indicated that the occurrence of citrulline on specific arginine residues in histones correlated with a decrease in the observed levels of methylation [52,53,125]. However, experiments performed *in vitro* cast some doubt on whether PADs truly possessed demethyliminase activity, since histone peptide substrates containing meR1, meR2s and meR2a were converted to citrulline at rates several thousand times slower than those observed for peptidyl arginine, or prevented citrullination entirely [159–161]. These data would suggest that methyl-arginines do not represent preferred substrate targets, and that methylation most likely antagonizes citrullination.

### (b) Cross-talk between citrullination and arginine methylation on histones

Several arginine residues on histone subunits are known to be a target for both methylation and citrullination. For example, PAD4 has been demonstrated to deiminate R2, R8, R17 and R26 in histone 3 and R3 in histone 4 [52,53]. In particular, levels of PAD4 have been shown to rise in response to oestrogen, and it can associate with the promoters of genes such as the oestrogen-responsive *pS2* where it targets the citrullination of H3 and H4 [52,53]. The appearance of the citrulline mark coincides with the disassociation of RNAP2 from the promoter and results in transcriptional repression of the gene [53]. Methylation of the *pS2* promoter on H3R17 and H4R3 by PRMT4/CARM1 and PRMT1 is also regulated in response to hormone induction but conversely, methylation occurs at earlier timepoints when RNAP2 is still engaged at the promoter [127,131,132]. This suggests that both arginine methylation and PAD4-targeted deimination are involved in oestrogen-mediated signalling, but have different temporal responses and opposing effects on gene expression.

Another example of citrulline–methylation interplay on histones is provided by examining the regulation of gene expression during haematopoiesis. T-cell acute lymphocytic leukaemia protein 1 (Tal1) is a transcription factor connected with the differentiation of monocytes and osteoclasts, and its deregulated activity is associated with haematological malignancies such as leukaemia [133]. Tal1 was shown to form a protein complex with the PAD4 enzyme, and can mediate its recruitment to a number of target genes involved in leucocyte differentiation such as interleukin 6 signal transducer (*IL6ST*) and CCCTC-binding factor (*CTCF*) [133]. On the *CTCF* promoter, PAD4 acts as a repressor by counteracting the active H3R17me2a mark generated by PRMT4/CARM1, while on the *IL6ST* promoter PAD4 acts as a coactivator by counteracting the repressive H3R2me2a mark created by PRMT6 [133]. There was also an observable interplay between PAD4 levels and the status of H4R3me2a at these promoters, which is another site known to be targeted by PAD4 that is mutually exclusive with arginine methylation [133].

The p53-target gene, *OKL38* (ovary, kidney and liver protein 38), which plays a role in cell growth inhibition and tumourigenesis, also appears to be regulated by the competitive activity of PADs and PRMT enzymes, which both modify the same target arginine residues on histones in the promoter of the gene, particularly H3R17 [58]. *OKL38* expression was induced after DNA damage in a number of cell lines and led to the induction of apoptosis in a p53-dependent manner [58]. p53 binding to the *OKL38* promoter was observed as early as 2 h after the induction of DNA damage, and its recruitment to promoter regions was coincident with increased levels of H3R17 methylation. By contrast, at the same timepoint there was a notable decrease in H3 citrullination marks, which correlate with the disassociation of PAD4 from the *OKL38* gene promoter [58]. H3 methylation, therefore, appeared to be associated with active transcription of the gene, while citrullination was correlated with gene silencing. A similar mechanism has also been described for other p53- and PAD4-regulated genes, including *p21*, *GADD45* and *PUMA* [59].

### (c) Cross-talk between citrullination and arginine methylation on non-histone proteins

A large number of non-histone proteins have been identified as targets for citrullination [162], and cross-talk between citrulline and arginine methylation on these proteins can often be important in regulating their function. For example, hormone-activated nuclear receptors (NRs) act as important transcription factors that integrate environmental and hormone signals to influence gene expression and cell fate decisions, and their activity is often deregulated in cancer [163]. They activate transcription by recruiting a number of coactivator proteins that help to remodel chromatin and assemble the RNAP2 transcriptional complex at the promoters of genes [163]. These coactivators include proteins such as glucocorticoid receptor interacting protein (GRIP1), p300/cAMP response element binding protein (CREB) binding protein (CBP) and the arginine methyltransferase PRMT4/CARM1, which can direct PTMs onto histones and non-histone proteins in the transcriptional complex [57]. Indeed, PRMT4/CARM1 can mediate the methylation of a C-terminal arginine residue of both p300 and CBP, which lies within a domain of the protein involved in interactions with GRIP1 [57]. Methylation by PRMT4/CARM1 was shown to inhibit the interaction between p300 and GRIP1, with a consequent reduction in the ability of the coactivator complex to induce the expression of a reporter gene [57]. PAD4 was subsequently shown to also target the same arginine residue on p300 and could effectively counteract the methylation event mediated by PRMT4/CARM1. As expected, PAD4, therefore, promoted interaction between p300 and GRIP1, and the interplay between methylation and deimination of this arginine residue contributed to the dynamic regulation of transcriptional control of NR-responsive genes [57].

Splicing factor proline- and glutamine-rich (SFPQ) protein is a highly abundant, multi-functional protein that acts in spliceosome assembly and interactions with other splicing components and pre-mRNA [164]. Alternative splicing is often deregulated in human cancers, and SFPQ itself has been implicated in prostate and breast cancers [165,166]. Like many other RNA-binding proteins (RBPs), SFPQ can be a target for arginine methylation, with a number of asymmetric methylation events on the protein being mediated by the PRMT1 enzyme [136]. These methylation events affect the efficiency with which SFPQ can associate with mRNA, and many of the arginine residues targeted for methylation can also be citrullinated by PAD4 in cells. Indeed, experiments with recombinant SFPQ and SFPQ-derived peptides indicated that prior citrullination by PAD4 could block subsequent methylation by PRMT1, and *vice versa*, and the balance between citrullination and methylation events may determine both the protein levels of SFPQ in cells and its ability to associate with mRNA [136] (figure 3*b*). It is proposed that this cross-talk between methylation and citrullination may, therefore, act as a cellular mechanism to post-translationally regulate RNA splicing and messenger ribonucleoprotein (mRNP) dynamics [136].

The cross-talk between methylation and citrullination on RBPs is not limited to SFPQ. The human ribosomal protein S2 (RPS2), which has been implicated in multiple types of tumours [137], contains an N-terminal arginine and glycine-rich repeat region targeted by PAD4 for citrullination [137] and PRMT3 for methylation [138–140]. These repeats of RG and RGG sequences are often collectively referred to as the RGG motif and have been discovered in more than 1000 human proteins. These RGG motif-containing proteins are implicated in a range of cellular pathways including transcription, RNA splicing, translation and DNA damage signalling [114,153,167–170]. The RGG motif is reported to play a key role in regulating protein–protein or protein–nucleic acid interactions [171,172]. The arginine residue in the RGG motifs is known to be a substrate for PRMTs, and as such, arginine methylation is thought to play an important regulatory role for RGG motif-containing proteins through modulating their interactions with other proteins or RNA. Indeed, given that arginine methylation seems to predominantly target RBPs, regulation of protein–RNA interactions may be a particularly important function of arginine methylation [141–147]. Arginine methylation is believed to prevent hydrogen bonding between RBPs and RNA through steric hindrance [148]. For example, PRMT4/CARM1-dependent arginine methylation of the RBP HuD is reported to reduce its binding affinity to *p21* mRNA [149]. On the other hand, this PRMT member is also known to methylate the RBP HuR, which in turn stabilizes its target SIRT1 mRNA [150]. This positive regulation of protein–RNA interactions is probably mediated by the resulting increased hydrophobicity of the arginine residue after methyl group addition [148].

Not surprisingly, RBPs containing RGG motifs can also be citrullinated by PADs. One proteomic study, which identified around 50 proteins as targets of PAD2 citrullination in cells, demonstrated that almost half of these citrullinated proteins are related to RNA splicing or processing [151]. The citrullinated RNA processing factors include some known RGG motif proteins such as hnRNPA1 and DDX2 [151]. In addition, another proteomic study identified more than 100 PAD4 substrates and approximately 20% of them were found to contain RGG motifs [152]. Moreover, it was revealed that PAD4 can competitively inhibit the methylation of some RGG motif proteins including the FET proteins (FUS, EWS and TAF15) and hnRNPA1, and each has been linked to amyotrophic lateral sclerosis (ALS) [152]. Indeed, PAD4-mediated citrullination was shown to reduce protein interactions and aggregation of these proteins [152], indicating a potential role for citrullination–methylation interplay in neurodegenerative diseases.

Another example of citrullination–methylation interplay and its potential involvement in RNA processing is found with the transcription factor E2F1 (figure 3*c–e*). The E2Fs are a family of transcription factors involved in diverse biological processes regulating numerous cell fate decisions in the cell. Their activity is primarily regulated by interactions with the retinoblastoma tumour suppressor protein (pRb); the pRb–E2F pathway is central in regulating cell cycle progression and its functional deregulation is of primary importance in proliferative diseases such as cancer [173]. E2F1 is perhaps the most studied family member and it is subject to several forms of PTMs that help dictate the different biological outcomes of E2F1 activity [174].

E2F1 possesses an RGRGR sequence, which is a target for residue-specific arginine methylation by PRMT5 and PRMT1. The balance of activity between these two enzymes can channel E2F1 activity into distinct biological pathways [114,153]. Thus, PRMT1-mediated methylation on E2F1 residue R109 augments E2F1-dependent apoptosis (figure 3*c*), while methylation by PRMT5 at residues R111 and R113 favours cell proliferation (figure 3*d*) [114,153]. Methylation by PRMT1 coincides with the ability of E2F1 to prompt apoptosis in DNA damaged cells, and acts to hinder methylation by PRMT5. By contrast, in cycling cells cyclin A binding to E2F1 impedes PRMT1-directed methylation and augments methylation by PRMT5, thus ensuring E2F1 is locked into its cell cycle progression mode [114,153]. This switch in biological output is mediated in part by influencing the recruitment and activity of E2F1 on subsets of target genes in response to PRMT1- or PRMT5-mediated methylation. In addition, the recruitment of a ‘reader’ protein, p100/Tudor staphylococcal nuclease (TSN), to PRMT5-methylated E2F1 acts to downregulate its apoptotic activity (figure 3*d*) [114,153].

Rather interestingly, E2F1 was also shown to interact with PAD4 in inflammatory cells, and this interaction was important for the deimination of the R109 residue targeted by PRMT1 [56]. Citrullination increased E2F1 transcriptional activity and promoted its recruitment to E2F target genes, specifically to cytokine genes involved in the immune response and inflammation [56]. The interplay between citrullination and methylation on E2F1, therefore, seems to be particularly important in directing its transcriptional activity towards particular subsets of genes, and in part helps to explain how E2F1 can influence such diverse sets of genes in response to varied biological stimuli.

More recently, the PRMT5-mediated arginine methylation event on E2F1 has been connected with the regulation of a diverse group of genes at the level of alternative splicing, with this role requiring the p100/TSN reader protein, which acts to recruit a large group of RNAs and components of the splicing machinery to E2F1 (figure 3*d*) [175]. Given the interplay between PAD4 citrullination and PRMT1- and PRMT5-directed methylation at the arginine-rich motif in E2F1, it seems likely that PAD4 activity will also regulate E2F1-dependent splicing events in cells, and this is an area of active current research.

Here, we have provided a few examples of proteins targeted by methylation/citrullination cross-talk to highlight the importance of the interplay between these two events, especially in the regulation of some RBPs and proteins involved with RNA splicing. Moreover, a collection of recent mass spectrometry studies have suggested that modulation of protein–RNA interactions by PRMTs and PADs could expand the role of these enzymes into the regulation of alternative splicing and RNA processing in cells [141–147,151,152] Citrullination of arginine residues directly removes their positive charge, thus one can expect that it may have a more direct, pronounced influence on the interaction with nucleic acids as compared to arginine methylation (figure 1). This may shed light on the possibility of PADs acting as a novel therapeutic target in diseases typified by aberrant RNA splicing, including many types of cancer.

### (d) Citrullination and other post-translational modifications

Despite the obvious potential for interplay between citrullination and methylation on the same target arginine residues, citrullination can also be integrated with other PTMs including acetylation, phosphorylation and lysine methylation [134,135,176]. Such cross-talk can be mediated due to the close proximity of the two modifications, or involves the recruitment of reader proteins or other accessory factors that regulate the generation of subsequent marks. Below we outline some examples of cross-talk between citrullination and other modifications on both histone and non-histone targets.

### (e) Cross-talk between citrullination and other post-translational modifications on histones

A number of arginine residues on histones are known to be deiminated by PAD2 and PAD4, and such modifications can have a downstream impact on other post-translational events. A good example is highlighted by the PAD2 enzyme, which is highly expressed in estrogen receptor (ER)-positive breast cancer and contributes to cell proliferation by regulating the expression of ER-target genes [81]. PAD2 mediates the citrullination of H3R26, and this modification occurs on the promoters of a number of ER-responsive genes and drives their expression [80]. The neighbouring lysine residue, H3K27, is also a site of multiple modifications, with lysine methylation being particularly important for gene expression regulation. For example, enhancer of zeste homologue 2 (EZH2) can target this site with a repressive methylation mark, which has been linked with numerous cancers [134]. It was discovered that H3R26 citrullination reduced the activity of EZH2 against H3K27 over a 1000-fold, while the H3K27 di- and tri-methyl mark could effectively inhibit citrullination of H3R26 by PAD2 [134]. Structural modelling predicted that the presence of K27me3 would reduce the binding interaction between H3 and PAD2, while conversely, citrullination of R26 by PAD2 was predicted to cause a conformational change in H3 that would preclude its methylation by EZH2 [134]. This interplay was recapitulated in cells where knockdown of one enzyme could result in an increased level of the alternate modification. This appears to be mediated in part by the PAD2-dependent recruitment of the H3K27 demethylase enzymes UTX and JMJD3, which are believed to facilitate the demethylation of H3K27 in cells [134].

Cross-talk between lysine methylation and citrullination can also have an impact on the recruitment of other ‘reader’ proteins to chromatin, which is exemplified in patients suffering from multiple sclerosis (MS), who show abnormal expression of a subset of cytokine genes including *TNFα* and *IL8* [135]. This abnormal expression is believed to be mediated as a result of decreased binding of the transcriptional repressor HP1*α* to the H3K9me3 chromatin mark at the promoters of these genes. It was demonstrated that PAD4, which is suspected to play a prominent role in MS [177–179], weakens the binding of HP1*α* to chromatin by deiminating the neighbouring H3R8 residue. This hypothesis is supported by the observation that peripheral blood mononuclear cells (PBMCs) taken from MS patients displayed defective HP1*α* recruitment to the *TNFα* promoter, while also displaying an accumulation of H3 with the double R8cit, K9me3 modification [135].

H3 citrullination has also been connected with the establishment of naive pluripotency during embryonic development and cellular reprogramming [176], and the presence of this modification is closely associated with the expression of SMARCAD1 (SWI/SNF-related, matrix-associated actin-dependent regulator of chromatin, subfamily A, containing DAED/H box 1), a DEAD/H ATP-binding protein that displays increased expression in developing mouse and human embryos, as well as being highly expressed in embryonal, mammary and lymphoid tumours [176]. SMARCAD1 appears to possess a binding specificity towards H3 peptides containing R26 citrulline, though it also has some preference towards acetylation of H3K27 too. H3K26cit marks exhibited extensive genome-wide co-localization with SMARCAD1 binding, as measured by ChIP-seq in mouse embryonic stem cells (ESCs), and SMARCAD1 knockdown caused ESCs to lose naive state features while remaining pluripotent [176]. Interestingly, SMARCAD1 knockdown led to the specific increase in the H3K9me3 mark at SMARCAD1-binding regions of the genome, and this was also evident in cells treated with PAD inhibitors, where there was an increase in H3K9me3 ChIP-seq signals at peak locations shared with H3R26cit ChIP-seq. This suggests that the recruitment of SMARCAD1 to H3R26cit in cells guards naive pluripotency by suppressing the H3K9me3 mark, although the precise mechanism of this interplay is not known at this time [176].

### (f) Cross-talk between citrullination and other post-translational modifications on non-histone proteins

Interactions between citrullination and other modifications are not restricted solely to histones, and many of the non-histone substrates for PADs are known to be regulated in a coordinated fashion by additional modifications. One example is the erythroblast transformation-specific (ETS)-like protein-1 (ELK-1) transcription factor, a member of the ternary complex factors family of ETS domain transcription factors, which are required for the regulation of many immediate-early genes in response to growth factor stimulation. ELK-1 is activated in response to MAPK/ERK signalling, and phosphorylation of ELK-1 leads to enhanced association with the p300 acetyltransferase, with subsequent induction of acetylation of target genes and enhanced gene activation [154]. PAD4 ChIP-chip experiments in MCF-7 breast cancer cells indicated that PAD4 could be associated with a large set of actively transcribed genes, and many of these gene promoters were enriched in the ELK-1 binding site motif. Indeed, PAD4 was shown to interact with ELK-1 in cells, particularly after stimulation with growth factors such as EGF, and both PAD4 and ELK-1 could be recruited to the promoter region of the immediate-early oncogene *c-Fos* [55]. ELK-1 was subsequently shown to be a direct substrate for PAD4 *in vitro*, and its citrullination promoted the subsequent ERK2-mediated phosphorylation of ELK-1, which was correlated with increased H4 acetylation and transcriptional activity at the *c-Fos* promoter [55]. Furthermore, PAD4 can modulate the TGF-β signalling pathway via citrullination of glycogen synthase kinase-3b (GSK3b) in MCF7 breast cancer cells [155]. Citrullinated GSK3b is sequestered away from the nucleus and thus results in an increase in TGF-β signalling [155]. Since this signalling pathway is a critical inducer of the epithelial-to-mesenchymal transition (EMT), it suggests that dysregulation of PAD4 may promote metastasis in cancer progression.

As discussed previously, E2F1 was also described as a non-histone target of citrullination by PAD4, and there is evidence of mutual exclusivity between citrullination and arginine methylation mediated by PRMT1 and PRMT5 [56]. Competition between citrullination and arginine methylation is not the only example of cross-talk on the E2F1 protein however, since citrullination has also been indicated to impact protein–protein interactions between E2F1 and BRD4, a member of the BET-family of bromodomain-containing readers that, like E2F1, is also implicated in modulating the expression of genes involved in the inflammatory response [56]. While the interaction of E2F1 with BRD4 is primarily dependent on the presence of acetylated lysine residues in E2F1, it was discovered that citrullination of flanking arginine residues enhanced the interaction between BET bromodomains and E2F1 peptides (figure 3*e*). In addition, treatment with a PAD4-specific inhibitor in cells reduced the levels of chromatin-bound E2F1–BRD4 complex at the promoters of inflammatory genes such as *TNFα*, leading to reduced expression of the cytokine [56]. In a murine CIA model, small molecule inhibition of PAD4 and BRD4 reduced the expression of inflammatory cytokines and was efficacious in preventing disease progression [56].

Cross-talk between citrullination and other modifications is not only restricted to interplay within a protein molecule, since citrullination of one protein can impact on the subsequent modification of another. For example, while screening for potential novel non-histone substrates of PAD4 in cells, inhibitor of growth protein 4 (ING4) was identified as a potential target. This protein is of particular interest since, just like PAD4, it is known to regulate histone modifications and can also interact with and modulate the activity of the p53 tumour suppressor protein [156]. ING family members are implicated in the suppression of initiation and progression of cancers in various tissues by their ability to promote cell cycle arrest, apoptosis and senescence. They also function in other cellular pathways such as the DNA damage response, cellular migration and invasion [156]. ING4 is known to enhance p53 transcriptional activity, in part by promoting acetylation of p53 and hence inducing downstream target gene expression [157]. ING4 was found to be citrullinated by PAD4 at arginine residues present within its NLS domain, which happens to be the domain of the protein involved in mediating the interaction with p53 (figure 3*f*). Indeed, citrullination of the ING4 NLS disrupted interactions with p53, and this resulted in reduced levels of p53 acetylation. Since ING4 was shown to be important for the recruitment of the p300 acetyltransferase to p53, it is thought that citrullination of ING4 and its subsequent loss of affinity for p53 would mediate the observed reduction in p53 acetylation [157,158].

## 4. Drug discovery and peptidylarginine deiminases

The regulation of PADs is closely associated with cancer progression, highlighting the potential of the protein family to be an attractive target for therapeutic intervention. Indeed, the development of PAD inhibitors has been a progressing area of clinical research for the past decade [73,180,181] and several PAD4-specific inhibitors were reported to have a promising anti-tumour effect in pre-clinical models [37,73,181–186]. GSK484, which is a selective reversible inhibitor of PAD4 widely used to study its enzymatic activities both *in vitro* and *in vivo* [73], was recently shown to sensitize triple-negative breast cancer cells to radiotherapy in a mouse xenograft model [182]. In addition, a more recent study demonstrated that the small molecule PAD4 inhibitor JBI-589 can suppress primary tumour growth in mouse models by preventing neutrophil accumulation in the tumour microenvironment via downregulation of chemokine receptor CXCR2 expression [183]. The irreversible pan-PAD inhibitor Cl-amidine is perhaps the most widely used compound to study the role of PAD enzymes in normal and pathological states, and has been shown to ameliorate disease severity in a number of animal studies, including mouse models for breast cancer, prostate cancer and glioblastoma [37,181,184–186]. However, the future success of PAD inhibitor development still requires a deeper understanding of PAD biology and the physiological impact of citrullination in cancer cells.

First, it will be essential to further analyse the cell ‘citrullinome’ in different disease conditions. Despite recent advances in the techniques used for the identification of citrullination by mass spectrometry [151,152], the numbers of proteins identified as being targets for citrullination are still more limited as compared to other PTMs [187]. Identification of novel PAD substrates will help us to understand the cellular pathways regulated by PAD activity in both healthy and diseased cells, which could potentially highlight novel therapeutic targets or biomarkers.

Second, it is important that future research investigate the cross-talk between PADs and other PTM-generating enzymes, which could open up the potential for combination drug therapy regimes. Current findings already suggest a promising possibility, considering that many RNA processing factors are substrates for both citrullination and methylation [141–147,151,152]. Splicing is commonly deregulated in cancers, either via mutation in RNA splicing factors, changes in the expression of splicing regulators or alterations to transcription factors that influence the process of splicing [188–195], and such tumours are particularly sensitive to therapeutic inhibition of splicing [196–199]. For example, SRSF2-mutant leukaemia cells are more sensitive than wild-type cells to inhibitors targeting components of the splicing network, such as the SF3B complex [147,196]. Moreover, inhibitors of symmetric (PRMT5-mediated) or asymmetric (type I PRMT-mediated) arginine methylation were also observed to preferentially kill serine and arginine-rich splicing factor 2 (SRSF2)-mutant leukaemia cells both *in vitro* and *in vivo*, and the mechanistic basis for this response in part reflects the observation that PRMTs predominantly target RBPs with established roles in splicing regulation [147]. Interestingly, combining PRMT inhibitors with other compounds targeting the splicing network (such as SF3B inhibitors) significantly reduced tumour growth in mouse models [147], suggesting that co-treatment of cancers with agents that mechanistically target distinct components of the splicing machinery could be used to control cancer cell proliferation and survival. Given that numerous RNA-binding proteins have recently been identified to be targets for both arginine methylation [56,134,135,152,175,176] and PAD-mediated citrullination [157,158], one predicts a similar synergistic effect may be observed in tumours co-treated with PAD and PRMT inhibitors. There may, therefore, be a future therapeutic benefit to consider combining current or newly developed PAD inhibitors with PRMT inhibitors already under clinical trials—for example, the PRMT5 inhibitor GSK3326595 (NCT03614728: https://clinicaltrials.gov/ct2/show/NCT03614728) [200,201], and type I PRMT inhibitor GSK3368715 (NCT03666988: https://clinicaltrials.gov/ct2/show/NCT03666988). Of course, an advanced understanding of both the molecular mechanism and biological consequences of PTM interplay is crucial here. Information such as the expression and target preferences of each enzyme needs to be collectively analysed, and the identification of proteins acting as ‘readers’ or ‘erasers’ of citrullination and other PTM marks will also add further complexity to the dynamic function and relationship between different PTMs.

In addition, there is growing interest in citrullinated proteins acting as neo-antigens, based on recent evidence suggesting that citrullinated peptides derived from target proteins can be presented by MHC class II molecules on the cell surface and elicit a CD4+ T-cell response [202]. For instance, one study demonstrated that immunization of mice with citrullinated peptides derived from alpha enolase led to increased survival rates and a strong CD4+ T-cell response in tumour models of melanoma, pancreatic carcinoma and lung carcinoma [203]. Another study indicated that immunization of mice with citrullinated vimentin peptides resulted in anti-tumour effects against subcutaneous B16 melanoma, inducing a CD4+ T-cell response and resulting in an approximate 80% survival rate [204]. Furthermore, a recent study revealed that immunizing with a combination of citrullinated enolase and vimentin peptides could stimulate strong CD4+ T-cell responses and potent anti-tumour effects, which may prevent tumour antigen escape [205]. Additionally, proteomic analysis in breast cancer patients has highlighted a strong positive correlation between PAD2 expression and a B-cell immune signature, as well as with MHC class II-bound citrullinated peptides [206]. These findings collectively emphasize the significance of citrullinated peptides as potential cancer vaccines and their possible use for diagnostic purposes. Importantly, vaccination with citrullinated peptides might be valuable for treatment of tumours with a low mutational burden, which tend to have fewer immunogenic neo-antigens. Further research in this area, coupled with the development of methods to identify immunogenic post-translationally modified epitopes, will be required to fully exploit the potential for citrullinated peptide vaccines to be used as immunotherapeutic agents.

Lastly, the efficacy of existing PAD inhibitors in human patients has yet to be confirmed, and the successful development of specific PAD inhibitors suitable for use in humans remains to be achieved. PADs potentially have opposing pro- and anti-cancer activities in cells, influenced by context- and tissue-dependent factors [37,40–42,84,86,87]. In addition, PADs have multiple activities in cells and are involved in a multitude of cellular pathways [52,207–211]. Continued effort towards the development of isoform-specific, clinically safe PAD inhibitors will, therefore, be imperative to cultivate powerful therapeutic strategies that can take advantage of any therapeutic window that exists between tumour and healthy cells.

## 5. Conclusion

Citrullination has recently emerged as a key PTM in multiple cellular processes and is implicated in many pathological conditions including cancer; yet our understanding of the underlying molecular mechanisms remains limited. In this review, we have summarized recent findings regarding the functions of citrullination that impact numerous cellular pathways in the course of tumour development. In addition, we have highlighted the interplay between citrullination and other protein modifications, leading to altered protein–protein or protein–nucleic acid interactions and their downstream influences on biological activities such as transcription and RNA processing. Extended analysis of citrullination and its interplay with other types of protein modification, coupled with more specific and powerful techniques for the manipulation and detection of PAD activities, are highly topical areas of current research, and continued future investigations in this field will hopefully highlight the potential for PAD family members to act as promising therapeutic targets.

## Data Availability

This article has no additional data.
